# Clinical utility of metagenomic next-generation sequencing in pathogen detection for lower respiratory tract infections and impact on clinical outcomes in southernmost China

**DOI:** 10.3389/fcimb.2023.1271952

**Published:** 2023-12-08

**Authors:** Jinxiang Hao, Weili Li, Yaoyao Wang, Jiangman Zhao, Yu Chen

**Affiliations:** ^1^ Department of Respiratory and Critical Care Medicine, Haikou Third People’s Hospital, Haikou, Hainan, China; ^2^ Zhangjiang Center for Translational Medicine, Shanghai Biotecan Pharmaceuticals Co., Ltd., Shanghai, China

**Keywords:** metagenomic next-generation sequencing, lower respiratory tract infection, diagnostic performance, antibiotic resistance genes, prognosis

## Abstract

**Background:**

Today, metagenomic next-generation sequencing (mNGS) has emerged as a diagnostic tool for infections. However, since Hainan has a complicated pathogen spectrum, the diagnostic value and impact on patient outcomes of mNGS in Hainan are to be explored.

**Methods:**

From April 2020 to October 2021, 266 suspected lower respiratory tract infections (LRTIs) patients in Hainan were enrolled, and specimens were collected before antibiotic treatment. Bronchoalveolar lavage fluid (BALF) samples were subjected to mNGS and culture to compare the diagnostic performance. Other conventional microbiological tests (CMT) were also performed. Patients’ treatments and clinical outcomes were recorded, and the antibiotic resistance genes (ARGs) were detected via mNGS workflow.

**Results:**

The positive rate of mNGS outperformed that of culture (87.55% vs. 39.30%, *p*<0.001) and CMT (87.12% vs. 52.65%, *p*<0.001). Specifically, mNGS detected more *P. aeruginosa* (12.03% vs 9.02%, *p*<0.05), *H. influenzae* (9.77% vs 2.26%, *p*<0.001), *Aspergillus fumigatus* (3.00% vs 0.75%, *p*<0.05), *Candida albicans* (26.32% vs 7.52%, *p*<0.001) and uncommon pathogens. It also demonstrated great diagnostic advantages in *Mycobacterium tuberculosis* with 80% sensitivity and 97.4% specificity. Over half of the patients (147, 55.26%) had modified empirical treatment according to mNGS results and 89.12% of them responded well. For three deaths with modified treatment, multiple drug resistance was predicted by mNGS and confirmed by antibiotic susceptibility test.

**Conclusions:**

The application of mNGS can benefit clinics in pathogen identification and antimicrobial treatment stewardship. Physicians should be alert to some emerging uncommon pathogens, including *Chlamydia Psittaci, Nocardia otitidiscaviarum*, and rare NTM.

## 1 Introduction

Lower respiratory tract infections (LRTIs), defined as pneumonia or bronchiolitis in the Global Burden of Diseases, Injuries, and Risk Factors Study, have been a leading cause of mortality and morbidity across the world (GBD 2016 Lower Respiratory Infections Collaborators., 2018). LRTIs can be caused by a wide range of pathogens but lack specific clinical manifestations in most cases, leading to many indefinite etiology diagnoses despite conventional diagnostic tests (Jain et al., 2015). Empirical broad-spectrum antibiotics are usually given to these patients to initiate early antibiotic treatment and timely appropriate empirical antibiotic treatment (EAT) is proven to improve clinical outcomes (Kollef et al., 2012; Zasowski et al., 2016). However, as a double-edged sword, EAT that is too broad also leads to abuses of antibiotics and selective resistance of pathogens (Jain et al., 2015), eventually impairing patients’ prognosis (Webb et al., 2019). What’s more, despite broad-spectrum antibiotics being prescribed, a large proportion of patients were still uncovered by the treatment (Miao et al., 2018). Therefore, timely pathogen identification is critical to tailored antibiotic treatment and to improve patient outcomes.

Culture is considered the gold standard for diagnosing bacterial infections (Charalampous et al., 2019), but it has evident limitations including time-consuming, limited sensitivity, and difficulty in detecting anaerobes (Carroll and Reimer, 1996; Eldin et al., 2019). The current culture-independent diagnostic methods include polymerase chain reaction (PCR) and serology tests, but they are targeted and need prior knowledge of the underlying pathogens. Metagenomic next-generation sequencing (mNGS) is an unbiased high-throughput sequencing technique (Lecuit and Eloit, 2015; Gu et al., 2019) that can screen out microorganisms without hypothesis in a short time. Besides, antibiotic resistance genes (ARGs) detection can also be integrated into its workflow (Simner et al., 2018; Serpa et al., 2022) for drug-resistance prediction. Today, mNGS has developed into a diagnostic tool for infectious and non-infectious diseases and abundant research has compared the diagnostic values of mNGS and culture. According to these studies, the sensitivity of mNGS in diagnosing pulmonary infections is superior to culture, which ranges from 44% to 97.2% (Wang et al., 2019; Zhou et al., 2019) according to different methodologies. However, recent research showed a geographic-location-heterogeneity in the pathogen spectrum of acute respiratory infections (Li et al., 2021; Qu et al., 2022), and it is under-investigated about the diagnostic value of mNGS for LRTIs in specific regions.

Hainan, the southernmost province of China, lies in the tropical region and has a tropical monsoonal climate. Due to the high temperature and intensive population movements of tourists, parasite endemics and viral pandemics, such as dengue, malaria, and COVID-19, severely affected this place (Xiao et al., 2010; Gao et al., 2022). The mobile population also leads to a broad and complicated pathogen spectrum in this place, where an etiological method that requires no prior hypotheses, such as mNGS, might be of capital importance to infection control in Hainan. Hence, the diagnostic performance of mNGS in such a place needs to be evaluated thoroughly. In addition, the clinical impact of mNGS on patients’ outcomes is seldom mentioned in previous studies. Therefore, we conducted a prospective observational study to find out the clinical utility of mNGS for LRTIs in Hainan, both in pathogen detection and in clinical outcomes. Also, antimicrobial resistance was analyzed via ARG detection in mNGS workflow among non-respondent patients.

## 2 Methods

### 2.1 Patients enrolment and data collection

**Figure 1:**
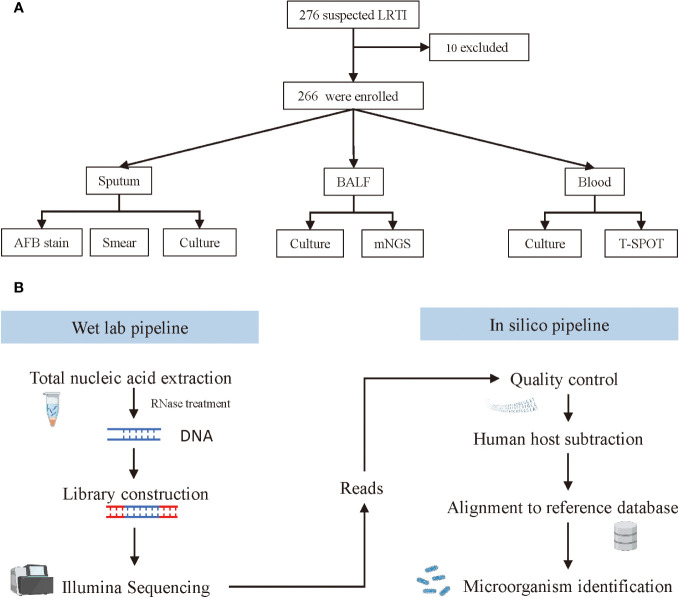
Screening, enrolment, and mNGS testing procedure of adult patients with LRTIs. **(A)** Flowchart of study design. **(B)** Schematic of mNGS sequencing and analysis.

From April 2020 to October 2021, patients suspected of lower respiratory tract infections (LRTI) were screened for SARS-COV-2, and SARS-COV-2 negative patients were enrolled at Haikou Third People’s Hospital in Hainan, China. The inclusion criteria were as follows: abnormal imaging findings, such as pulmonary shadows, space-occupying lesions, and other signs of pulmonary infection, combined with≥1 following items: 1) fever >37°C; 2) symptoms such as cough, expectoration, or dyspnea; 3) leukocytosis; 4) clinical signs of lung consolidation or moist rales. The exclusion criteria were: 1) refuse to participate in the study or fail to provide BALF samples; 2) recent antibiotic usage within a month. The study design was displayed in **Figure 1A**. The diagnosis of LRTI was made by two attending physicians based on a *post hoc* comprehensive approach which combined clinical adjudication and microbiological tests results. Baseline characteristics of all patients were collected on enrolment, including age, gender, temperature on admission, and underlying diseases. Blood routine tests results, C-reactive protein and procalcitonin levels, imaging findings and antibiotic regimens were recorded during hospitalization. The antibiotic treatment response, length of hospitalization and patient outcomes were also investigated. This study was performed in accordance with the Declaration of Helsinki. Ethics approval for this human study was approved by Haikou Third People’s Hospital Ethics Committee ([2020]K001). All adult participants or surrogates provided written informed consent to participate in this study.

### 2.2 Specimen collection

All patients received antibiotic treatment immediately after hospitalization. Before that, bronchoalveolar lavage fluid (BALF) specimens were collected with an ultrathin bronchoscope (BF-P-260F, Olympus), and then were divided into two parts and sent for culture and mNGS (Biotecan, Shanghai) in a pairwise manner. Blood samples and sputum samples were also collected simultaneously for conventional microbiological tests (CMT), including culture, smear, T.SPOT, and acid-fast bacteria stain (AFB stain).

### 2.3 DNA extraction, library construction, and sequencing

The workflow of mNGS sequencing consisted of a wet lab pipeline and *in silico* pipeline (**Figure 1B**). For the web lab workflow, microcentrifuge tubes containing 0.5 mL BALF sample and 1 g glass beads of 0.5 mm were first attached to a horizontal platform on a vortex and agitated vigorously at 2800-3200 rpm for 30 min. Then, separate 0.3 mL of the sample into a new 1.5 mL microcentrifuge tube, and extract DNA using a HostZEROTM Microbial DNA Kit (D4310, ZYMO RESEARCH) according to the manufacturer’s instructions. To prepare the library, more than 5 ng DNA extraction was digested into appropriate length (200–300 bp) followed by attaching the 3’ end with a Da tail and the 5’ end phosphorylation. Next, connect the fragmented DNA to the adapter sequence with DNA ligase, and remove the splice dimers, redundant splices, and residual reagents with purification beads. Finally, all sample DNA libraries with a concentration of more than 1 nmol/L were mixed and sequenced with an Illumina NextSeq CN500 sequencer (SE strategy, read length=75). In this study, internal control, negative control, and positive control were used for each run. Before nucleic acid extraction, specific molecular tags were placed in the sample and used as internal parameters to track the whole process and control the quality of the workflow. No-template water was applied as a negative control to monitor sample-to-sample contaminations. Tests should be repeated when the negative control failed. The clinical samples with confirmed pathogens served as a positive control, which indicated failures of mNGS when the targeted pathogens failed to be detected in these samples.

### 2.4 Bioinformatic analysis

For the *in silico* analysis, clean sequencing data were filtered with Fastp software (v0.23.1) by the following steps: 1) removing connector sequences from reads and reads that have no insert fragments due to connector self-connection; 2) removing low-quality (length <20 bp) reads at 3’ end; 3) removing reads that contain over 10% of N; 4) removing Adapter and sequences shorter than 15 bp after trimming. Seqtk_sdust (v1.3-r106) was utilized to calculate the complexity of each read and low complexity (<0.3) sequences were filtered out. By mapping the human host sequences to the human reference genome (grch38.p12) using bowtie2 (v 2.3.4.1), the remaining human sources of reads were removed. After removing the plasmid sequence and chromosome incompletely assembled sequence, species with completely assembled genomes and chromosomes were included in the pathogenic microorganism database. Then, map the microbial reads via bwa (v0.7.15) and classify pathogenic bacteria with whole genome alignment by Kraken software. The Pathogenic microbial genomic data originates from NCBI refseq (ftp://ftp.ncbi.nlm.nih.gov/genomes/refseq/) and PATRIC (https://www.patricbrc.org/). Finally, all detected pathogens were calculated for alignment sequence number, relative abundance, and genome coverage.

For Antibiotic Resistance Genes (ARGs) analysis, the sequences of pathogens were aligned to the CARD (the Comprehensive Antimicrobial Resistance Database) database, an established drug resistance gene database, by blast (v 2.2.26). Sequences with over 90% of similarity identity and more than 70 bp in length were retained. The coverage of the ARGs was calculated according to the starting position of the comparison, and ARGs with coverage ≥80% were defined as positive.

### 2.5 Criteria for considering mNGS positive

First, bacteria suspected of colonizers were excluded referring to the in-house background microbial database. Then, the remaining bacteria (mycobacteria excluded), viruses, and parasites undergo the following filtering criteria:

1) Bacteria or virus: coverage rate scored 10-fold greater than that of any other microbes (Langelier et al., 2018);
2) Fungi: coverage rate scored 5-fold higher than that of other fungus (Schlaberg et al., 2017), or supported by clinical culture (Chen X. et al., 2020);
3) *Mycobacterium tuberculosis* (MTB): reads ≥ 1 (Langelier et al., 2018) since its DNA is hard to extract and unlikely to be contaminated for BALF samples; Nontuberculous mycobacteria (NTM) were defined positive when the reads ≥ 10 or ranked within the top 10 in the bacteria list (Schlaberg et al., 2017).

The responsible pathogens for LRTIs were assessed by two attending physicians in our hospital based on the microbiological results, clinical features, and response to the treatment. Treatment response was evaluated based on a comprehensive adjudication considering the patient’s symptoms, laboratory indicators, and radiological signs.

### 2.6 Statistical analysis

Normally distributed continuous variables are recorded as the mean ± standard deviation and nonnormally distributed were as the median and range. Categorical variables are reported as numbers and percentages. The McNemar-test was used for comparisons of the diagnostic performance between two diagnostic methods. For comparison of the clinical laboratory indicators before and after treatment, a paired t-test was applied. All tests were two-tailed, and statistical significance was set at *p*<0.05. SPSS software (version 27.0; IBM Corporation, Armonk, NY, USA) was used for the statistical analysis.

## 3 Results

### 3.1 Patient baseline characteristics

In this prospective observational study, a total of 276 patients suspected of LRTIs were screened. After excluding 10 cases, consisting of two non-infectious cases and eight repeated cases, there were 266 patients included for analysis. The demographic features of these patients were presented in **Table 1**. The average age of all patients was 67.25 years old. Among them, 177 (66.5%) were males and 89 (33.5%) were females. Patients with immunodeficiency accounted for 12.8%. There were 63 patients presented with fever, ranging from 37.2 °C to 40.8 °C. There were 136 patients accompanied with underlying disease, most of them were bronchiectasis (22.9%) and chronic obstructive pulmonary disease (COPD, 19.9%). Empirical antibiotics were given after BALF collection and before microbiological results.

**Table 1:** Demographic features.

	All patients (n=266)
**Age (yrs, mean ± SD)**	67.25 ± 16.32
Sex, n(%)	
Male	177 (66.5%)
Female	89 (33.5%)
Immunol deficiency, n(%)	
Yes	34 (12.8%)
No	232 (87.2%)
**Fever, n(%)**	
Yes	63 (23.7%)
no	203 (76.3%)
Underlying diseases, n(%)	
Asthma	7 (2.6%)
Bronchiectasis	61 (22.9%)
Pulmonary fibrosis	2 (0.0%)
Chronic obstructive pulmonary disease (COPD)	53 (19.9%)
Cardio cerebrovascular disease	6 (2.3%)
Chronic kidney diseases	1 (0.0%)
Malignancies	9 (3.4%)
Blood laboratory examination (range)	
White blood cell, 10^9^/L	9.89 (2.27-34.17)
Neutrophil, %	73.32 (10.5-94.9)
Hemoglobin, g/L	123.38 (67–182)
Platelet, 10^9^/L	262.53 (31–585)
C-reactive protein, mg/L	52.35 (0.05-274.4)
Procalcitonin, ng/mL (n=41)	3.23 (0.01->50)
Clinical outcomes	
In-hospital mortality, n(%)	6 (2.2%)
Length of hospital stay (d, mean ± SD)	8.48 ± 3.90

### 3.2 Diagnostic performance of mNGS and culture

**Figure 2:**
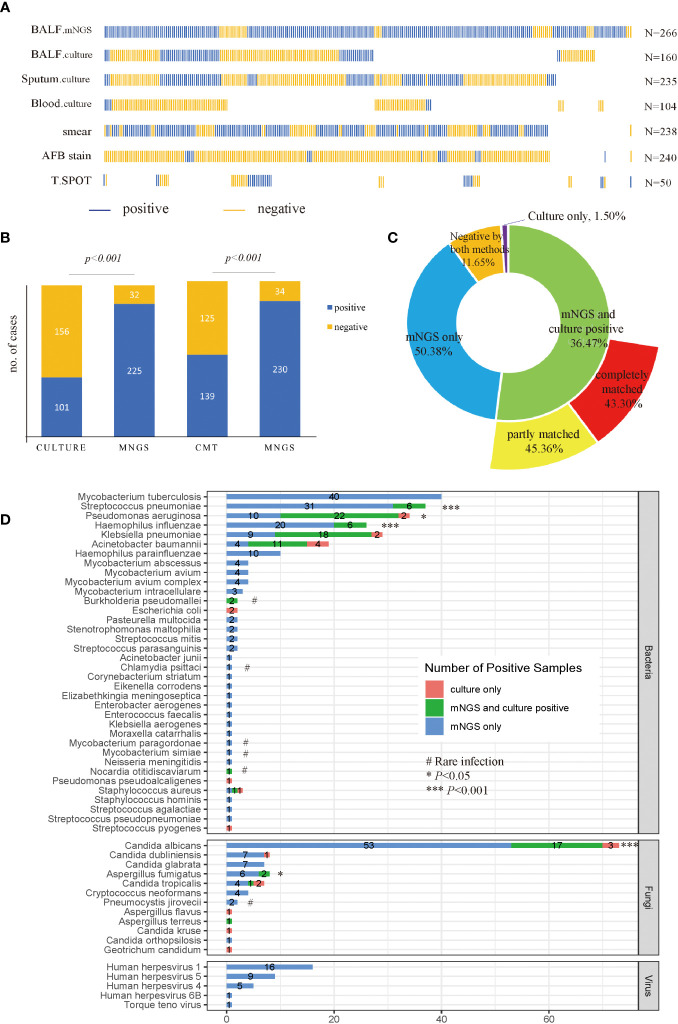
Sample distribution and diagnostic performance of mNGS and culture. **(A)** Distribution of samples. Each column of sticks represents a single patient. Blue and orange sticks represent positive and negative results, respectively; **(B)** Comparison of positive rates between mNGS and culture, and CMT, in a pairwise manner. **(C)** Concordance of mNGS and culture results. **(D)** Detecting performance at the pathogen level. The culture in **(B–D)** included BALF, sputum and blood culture results. Specifically, a positive culture result refers to at least one culture is positive, and a negative culture result means all cultures were negative. *p*-value was calculated with McNemar-test. *p<0.05, ***p<0.001; # rare infection.

BALF samples were collected from patients and divided into two parts for mNGS sequencing (n=266) and culture (n=160), respectively. Other samples were also collected for culture if needed, including sputum (n=235), and blood (n=104). Meanwhile, other conventional microbiological tests (CMT) were also performed with sputum samples and blood samples, including smear (n=238), AFB stain (n=240), and T. SPOT (n=50). The distribution of sample types and microbiological tests is shown in **Figure 2A**.

As shown in **Figure 2B**, among 257 patients with accessible culture results, the positive rate for mNGS (87.55%) was higher than culture (39.30%) (*p*<0.001). Likewise, among 264 patients who were tested by CMT, the diagnostic performance of mNGS was still higher than CMT (*p*<0.001), with a positive rate of 87.12% versus 52.65%. Among all patients enrolled, a total of 134 cases (50.38%) were positive for mNGS only, 97 cases were positive for both mNGS and culture, and only 4 cases were positive by culture only (**Figure 2C**). Among the 97 double-positive samples, 88.66% of them showed matched results between mNGS and culture completely (44 cases) or partly (42 cases). As for the 160 cases who had both mNGS and culture tests with BALF samples, the positive rate of mNGS was still superior to culture (88.75% vs 26.88%, *p*<0.001). A total of 43 cases (26.87%) were positive for both mNGS and culture, and 83.72% of them showed matched results between mNGS and culture completely (22 cases) or partly (14 cases), displaying a relatively high consistency between mNGS and culture results (**Supplementary Figure 1**).

### 3.3 Pathogens of LRTIs detected by mNGS and culture

A total of 208 microbes were isolated by mNGS, among which 36 bacteria, 12 fungi, and 5 viruses were considered underlying pathogens. For bacteria, MTB was the most detected pathogen (n=40), followed by *Streptococcus pneumoniae* (n=37), *Pseudomonas aeruginosa* (n=32), *Haemophilus influenzae* (n=26), *Klebsiella pneumoniae* (n=27), and *Acinetobacter baumannii* (n=15). The mNGS detected more pathogens than culture in terms of MTB (mNGS only), virus (mNGS only), *S. pneumonia* (13.91% vs 2.25%, *p*<0.001), *P. aeruginosa* (12.03% vs 9.02%, *p*<0.05), *H. influenzae* (9.77% vs 2.26%, *p*<0.001), *Aspergillus fumigatus* (3.00% vs 0.75%, *p*<0.05), and *Candida albicans* (26.32% vs 7.52%, *p*<0.001) (**Figure 2D**). Notably, in cases where culture was negative, mNGS showed advantages in detecting uncommon pathogens, including *Chlamydia psittaci* (n=1), *Cryptococcus neoformans* (n=4), *Pneumocystis jirovecii* (n=2), *Nocardia otitidiscaviarum* (n=1), *Mycobaterium paragordonae* (n=1) as well as *Mycobaterium simiae* (n=2). Besides, both mNGS and culture isolated *Burkholderia pseudomallei* from two patients.

Since all cultures failed to report MTB till the data was collected, the diagnostic performance for mNGS and other conventional methods, including T-SPOT and AFB stain, were compared with the final diagnosis of MTB. The final diagnoses of MTB were made by the attending physicians according to the China Clinical Diagnosis Guide for Tuberculosis (National Health and Family Planning Commission of the People’s Republic of China., 2017). The sensitivity of mNGS for diagnosing MTB was 80%, which was greatly higher than T-SPOT (28%, *P*<0.05) and AFB stain (26%, *P*<0.01) (**Table 2**). The diagnosing specificity of mNGS was 97.4%, similar to the T-SPOT (89.6%) and AFB stain (93.0%).

**Table 2:** Diagnostic performance of mNGS, T-SPOT, and AFB stain for MTB compared with the clinical final diagnosis.

	Sensitivity (95% CI; n/N)	Specificity (95% CI; n/N)
mNGS	80% (40/50) *^a,b^*	97.4% (112/115)
T-SPOT	28% (14/50) *^a^*	89.6% (103/115)
AFB stain	26% (13/50) *^b^*	93.0% (107/115)

p-values were calculated with McNemar-test. ^a^mNGS vs. T-SPOT p<0.05; ^b^mNGS vs. AFB p<0.01,

### 3.4 Antimicrobial treatment adjustment and clinical outcomes

**Figure 3:**
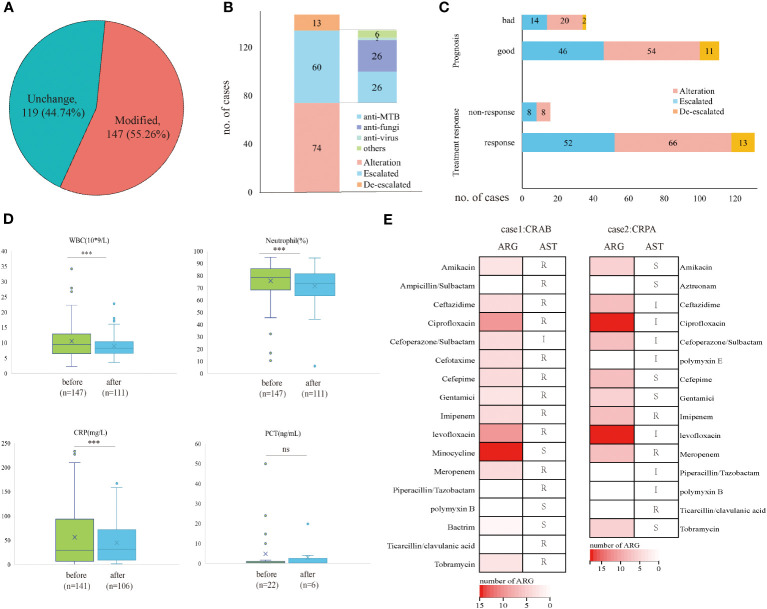
Clinical outcomes of mNGS-guided treatment stewardship. **(A)** Proportion of patients who modified antimicrobial regimens. **(B)** Distribution of detailed EAT adjustment strategies. **(C)** Clinical outcomes of patients with modified treatment regimens. **(D)** Laboratory tests change before and after treatment adjustment. **(E)** Number of ARG and AST results of CRAB patient and CRPA patient. *p*-value was calculated with a paired t-test. ***p<0.001; ns, not significant. R, resistant; I, intermediate; S, sensitive.

Based on the mNGS result, 147 (55.26%) cases modified the initial antibiotic treatment, including 74 cases of adjusted antimicrobial regimens, 60 patients escalated the treatment by increasing dosage or medication, and 13 cases de-escalated the EAT. Among 60 patients who escalated the antimicrobial treatment, 26 added anti-MTB meds, 26 escalated with anti-fungi treatment, and 2 with anti-virus treatments (**Figures 3A, B**). The treatment responses and prognosis of patients with mNGS-guided antibiotic adjustment were displayed in **Figure 3C**. There were 89.12% of patients responding to modified regimens and 75.51% of patients with good prognosis. After adjusting treatments guided by mNGS results, clinical laboratory indicators, including white cell counts (WBC), neutrophils (N), and C-reactive protein (CRP), declined remarkably when compared to baseline (all *p*<0.001) (**Figure 3D**). Notably, all 13 patients with a de-escalated adjustment also responded to treatment and the overall prognosis were well.

However, five patients died during hospitalization despite positive results by mNGS and culture and had modified antimicrobial treatment accordingly. They responded poorly to the antibiotic treatment, and three of them had identical mNGS and culture results. Therefore, the antimicrobial resistance of these patients was analyzed retrospectively by mNGS workflow and compared with the antibiotic susceptibility test (AST) if applicable. A total of 38 antibiotic-resistant genes (ARGs) were detected in a patient confirmed of *P. aeruginosa* infection (**Supplementary File 1**) and 25 ARGs in a patient confirmed of *A. baumannii* infection (**Supplementary File 2).** No ARG was detected in a patient confirmed of *C. albicans* infection. For the patient with *P. aeruginosa* infection, AST confirmed carbapenem-resistant *P. aeruginosa* (CRPA) infection and eight ARGs were predicted to be resistant to carbapenems and polypeptides, including ArmR, MexA, MexB, mexP, mexQ, mexY, opmE, and OprM. Besides, for the patient with *A. baumannii* infection, AST confirmed carbapenem-resistant *A. baumannii* (CRAB) infection, and six ARGs were detected to be resistant to carbapenems and cephalosporins, including adeJ, adeK, msrE, adeI, abeM and adeN (**Figure 3E**).

### 3.5 Image findings

**Figure 4:**
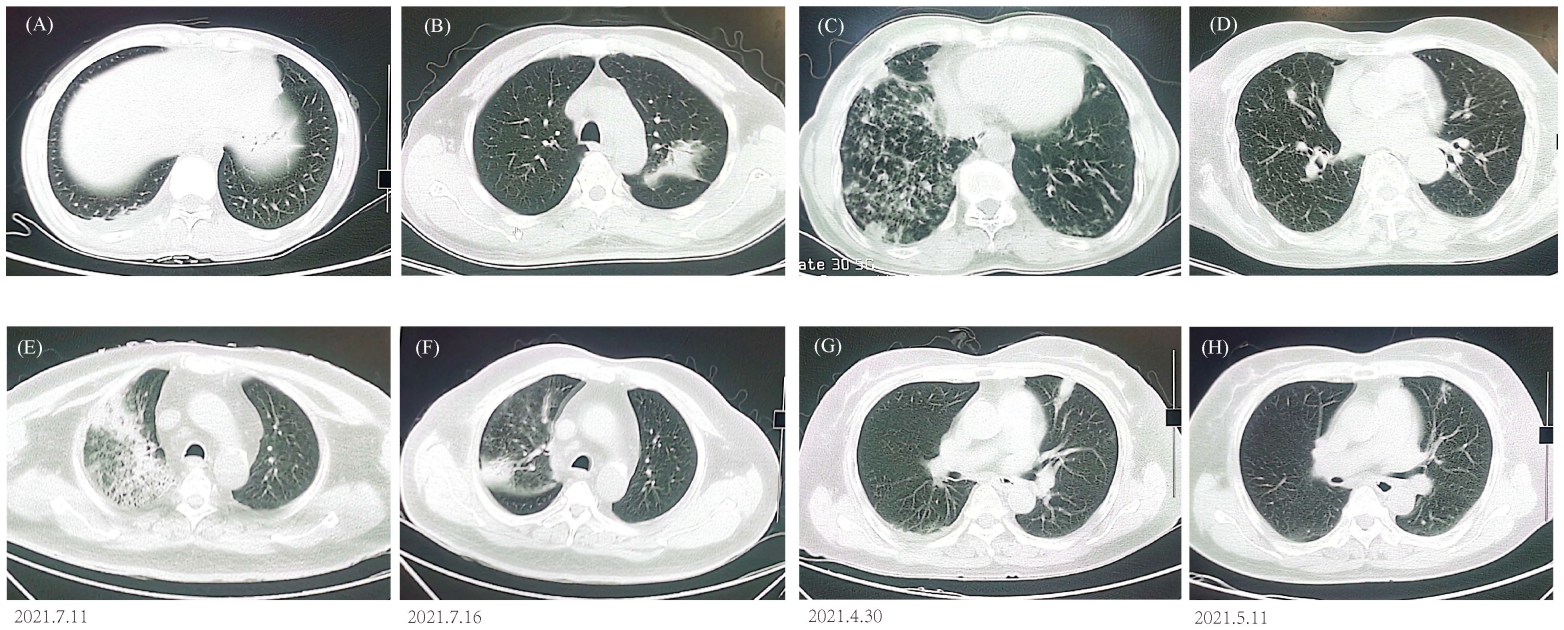
Chest CT findings. **(A)** *P. jirovecii* showed ground-glass opacity in lower right lobe. **(B)** *M. paragordonae* displayed patchy infiltration and consolidation in left lobe. **(C)** *N. otitidiscaviarum* with bilateral multiple patchy ground-glass opacities and exudated consolidations. **(D)** A mixed infection exhibited bilateral multiple ground-glass nodules. CT images of *C. psittaci* **(E, F)** and *C. neoformans* **(G, H)** before and after antibiotic adjustment.

All patients demonstrated abnormal chest CT images after enrollment, with 184 (69.17%) bilateral lesions and 82 (30.83%) unilateral lesions. Atypical pathogens and some less-common pathogens usually cause various appearances in CT scans (**Figures 4A–D**), but mNGS can detect unbiasedly and guide clinical treatment. A 63-year-old patient presented with cough, fever, shortness of breath, and multiple patchy rashes over the body. CT image indicated large consolidations in the upper right lobe and mNGS reported *Chlamydia psittaci* and MTB. The lesions were remarkably absorbed after antibiotic treatment based on mNGS results (**Figures 4E, F**). A 59-year-old patient had abnormal laboratory test results and bilateral multiple patchy consolidations in the chest CT scan. Empirical antibiotic treatment failed and was stopped after mNGS reported *Cryptococcus neoformans* infection, the patient was immediately changed into an anti-fungi regimen. Twelve days later, the laboratory indicators returned to normal, and the consolidated nodules shrunk obviously compared to that at hospitalization (**Figures 4G, H**).

## 4 Discussion

Numerous studies have reported the superiority of mNGS to culture in pathogen identification in infectious diseases for its higher sensitivity and unbiased broad detecting spectrum. In this study, we further explored the clinical impact of mNGS in Hainan as well as its extra potential in predicting antimicrobial resistance. Consistent with other studies (Chen H. et al., 2020; Liang et al., 2022), we found a superiority of mNGS in diagnostic performance for common community-acquired pneumonia (CAP) pathogens, including viruses, *S. pneumoniae, H. influenzae, and P. aeruginosa*. However, detection of common pathogens is far from enough for such a costly method as mNGS, and gratifyingly, it exhibited an extraordinary advantage in identifying MTB, NTM, fungus, and atypical and rare pathogens for suspected LRTIs patients in this study. Besides, thanks to the quick turnaround time of mNGS, over half of the patients timely shifted their initial empirical treatment to a more tailed regimen, and most of them responded well and turned better. This indicated that mNGS had a positive impact on patients’ outcomes.

Pulmonary tuberculosis (PTB) is a heavy burden globally with over 10 million estimated new cases all over the world (Bagcchi, 2023). China has the third largest PTB burden and Hainan is one of five provinces with the highest rate of reported PTB cases (Chen W. et al., 2020; Zhang et al., 2020). However, the clinical manifestations of PTB can be non-specific and signs of PTB often resemble those of malignancies or sarcoidosis (Skoura et al., 2015). Therefore, the diagnosis of PTB relies extensively on microbiological tests and clinical suspicion. Current microbiological methods for MTB include culture, smear, AFB stain, T.SPOT, interferon-gamma (IFN-γ) release assays (IGRAs) and PCR-based methods (Opota et al., 2019). But all these methods suffer many limitations, such as time-consuming, low sensitivity, and inability to differentiate MTB and NTM. Our study found that mNGS can detect *M. tuberculosis* with high sensitivity and specificity compared to AFB stain and T.SPOT, and can greatly shorten turnaround time compared to culture(2-3 days vs. 4-6 weeks). Therefore, mNGS is a promising technique for MTB detection in high MTB burden areas. Nevertheless, there were still 10 PTB cases that were missed by mNGS, which was probably because *M. tuberculosis* resides inside cells, making it difficult to extract the pathogen DNA. Apart from MTB, mNGS also detected 17 non-tuberculous mycobacteria (NTM) cases in this study, including two rare NTM pathogens, namely *M. paragordonae* and *M. simiae*. *M. paragordonae* was reported to be an emerging pathogen lately, with a lower than 0.5% isolation rate, and non-specific manifestations and CT signs (Li et al., 2022). In this study, however, the patient with *M. paragordonae* infection exhibited sporadic erythema and scleromas over bilateral palms and lower limbs, which can broaden current knowledge of the clinical symptoms of *M. paragordonae*.

Except MTB and NTM, mNGS identified uncommon pathogens in cases where culture yielded negative results, for example, *C. psittaci*, *P. jirovecii*, and *N. otitidiscaviarum*. The former two pathogens have drawn intensive attention from clinics for their increasing incident rates. However, the *Nocardia* genus can cause neglected but potentially life-threatening tropical diseases. Among over 200 *Nocardia* species, *N. otitidiscaviarum* is a less-common zoonotic pathogen and was first reported of a human infection in the 1960s (Mariat, 1965). In China, *N. otitidiscaviarum* was mainly reported in the eastern coastal provinces of China (Wang et al., 2023). In more recent years, fatal cases were reported of drug-resistant *N. otitidiscaviarum* infections (Barry et al., 2022; Fu et al., 2023). However, since most clinical and radiological signs of *N. otitidiscaviarum* resemble that of MTB and NTM (Duggal and Chugh, 2020), it is of capital importance to obtain a microbiological diagnosis for proper treatment. In this study, a 63-year-old female with COPD was hospitalized for pneumonia. After mNGS reported *N. otitidiscaviarum* infection, the patient was administered with sulfamethoxazole and turned well.

The diagnostic performance has been thoroughly discussed in multiple systems as well as in this study, but its impact on treatment strategies and clinical outcomes in real-world scenarios is less mentioned. Miao et al. (2018) found up to 60% of patients were uncovered by EAT and 37% of patients were given inappropriate treatment. Liang et al. (2022) reported over 80% of patients adjusted or confirmed antibiotic treatment based on mNGS. But neither of these studies mentioned the prognosis of these patients. In this study, over half of the patients modified the initial EAT regimen based on the mNGS results and most of them responded to the alterations. Since mNGS can identify novel variants of ARGs (Simner et al., 2018), we moved a step further to analyze the drug resistance for death cases. Two of the three cases were identified with ARGs, which indicated multidrug resistance. The AST results also proved the drug resistance, which might be the reason for the poor response to treatments and fatality of the two cases. However, the ARG analysis in this study was performed retrospectively in a small sample size, the prediction value of mNGS for drug resistance remains obscure and demands a larger cohort to evaluate it. Nonetheless, since AST can only be performed on cases with positive culture results, mNGS seems to provide extra drug-resistance information for culture-negative patients.

Our study has some limitations, too. First, there is no universal standard for positive criteria and results vary among different laboratories. Second, the cost of mNGS is high compared to conventional methods, therefore mNGS is especially recommended for critically ill patients where timely result outweighs the cost-effectiveness, and in patients where common pathogens were ruled out by conventional methods. Third, due to the limitations of our hospital, we didn’t perform MTB culture in parallel with mNGS, which otherwise could have compared the diagnostic performances between mNGS and MTB culture.

In conclusion, mNGS can be applied to clinical routines for severely ill patients and places with complicated pathogen spectrums. Also, patients’ outcomes guided by mNGS were excellent due to the optimized treatment strategy. Thanks to its superior sensitivity and quick turnaround time, mNGS is a good screening tool that can be used together with culture, but culture remains the gold standard for diagnostic purposes.

## Data availability statement

The datasets presented in this study can be found in online repositories. The names of the repository/repositories and accession number(s) can be found at: https://www.ncbi.nlm.nih.gov/bioproject/PRJNA938262/**Supplementary Material**.

## Ethics statement

The studies involving humans were approved by Ethics Committee of Haikou Third People’s Hospital. The studies were conducted in accordance with the local legislation and institutional requirements. The participants provided their written informed consent to participate in this study.

## Author contributions

JH: Conceptualization, Investigation, Methodology, Writing – original draft. WL: Data curation, Investigation, Visualization, Writing – original draft. YW: Formal analysis, Methodology, Software, Writing – original draft. JZ: Funding acquisition, Investigation, Supervision, Validation, Writing – review & editing. YC: Conceptualization, Data curation, Project administration, Resources, Supervision, Validation, Writing – review & editing.
