# Supplementary material for: Clinical utility of metagenomic next-generation sequencing in pathogen detection for lower respiratory tract infections and impact on clinical outcomes in southernmost China
Source: Front Cell Infect Microbiol. 2023 Dec 8;13:1271952. doi: 10.3389/fcimb.2023.1271952 (PMC10739398; doi:10.3389/fcimb.2023.1271952)
Supplement: Supplementary file 1 [file DataSheet_1.pdf]

Supplementary Material

Clinical Utility of Metagenomic Next-Generation Sequencing in Pathogen Detection for Lower Respiratory Tract Infections and Impact on Clinical Outcomes in Southernmost China.

Jinxiang Hao[1†], Weili Li[2†], Yaoyao Wang[2], Jiangman Zhao[2], Yu Chen[1*]

[1]Department of Respiratory and Critical Care Medicine, Haikou Third People's Hospital, Haikou, Hainan, China

[2]Zhangjiang Center for Translational Medicine, Shanghai Biotecan Pharmaceuticals Co., Ltd., Shanghai, China

[†]These authors contributed equally to this work and shared first authorship.

* **Correspondence:**

Yu Chen

chyy95@126.com

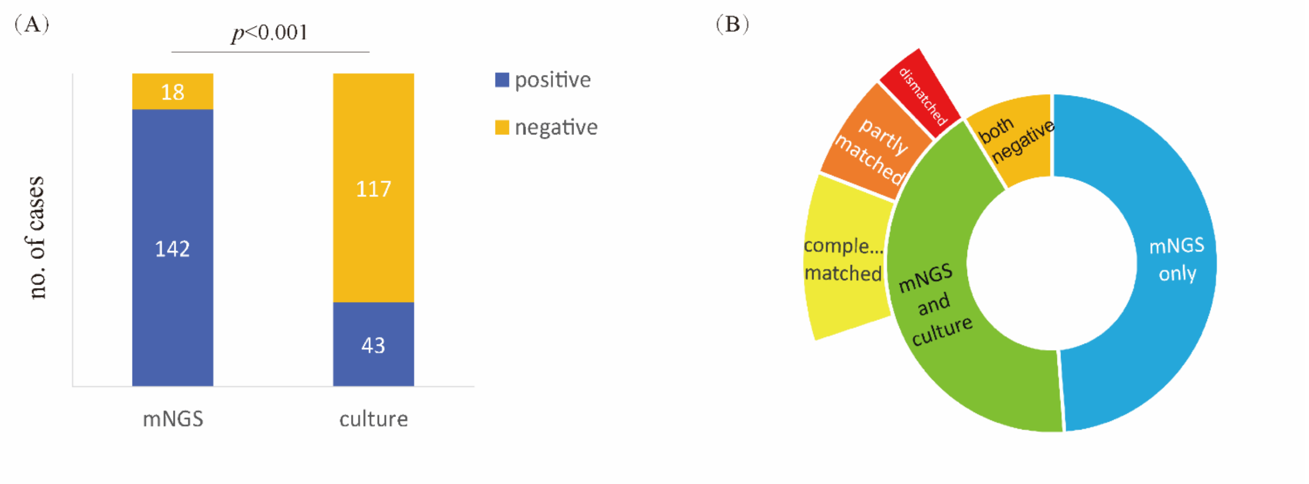

Supplementary Figure 1. Comparison of diagnostic performance of mNGS and culture with BALF samples (n=160). (A) Comparison of positive rates between mNGS and culture in a pairwise manner. (B) Concordance of mNGS and culture results.
